# Prophylactic Valproic Acid Treatment Prevents Schizophrenia-Related Behaviour in *Disc1*-L100P Mutant Mice

**DOI:** 10.1371/journal.pone.0051562

**Published:** 2012-12-18

**Authors:** Tatiana V. Lipina, Fahmida Nipa Haque, Alexander McGirr, Paul C. Boutros, Thorsten Berger, Tak W. Mak, John C. Roder, Albert H. C. Wong

**Affiliations:** 1 Samuel Lunenfeld Research Institute at Mount Sinai Hospital, Toronto, Ontario, Canada; 2 Centre for Addiction and Mental Health, Toronto, Ontario, Canada; 3 Informatics and Biocomputing Platform, Ontario Institute of Cancer Research, Toronto, Ontario, Canada; 4 Campbell Family Institute for Breast Cancer Research, Ontario Cancer Institute, University Health Network (UHN), Toronto, Ontario, Canada; 5 Departments of Medical Biophysics and Molecular and Medical Genetics University of Toronto, Toronto, Ontario, Canada; 6 Department of Psychiatry, Faculty of Medicine, University of Toronto, Toronto, Ontario, Canada; “Mario Negri” Institute for Pharmacological Research, Italy

## Abstract

**Background:**

Schizophrenia is a neurodevelopmental disorder with onset early in adulthood. Disrupted-In-Schizophrenia-1 (DISC1) is a susceptibility gene for schizophrenia and other psychiatric disorders. *Disc1*-L100P mutant mice show behaviors relevant to schizophrenia at 12 weeks, but not at 8 weeks of age, and may be useful for investigating the onset of schizophrenia in early adulthood.

**Methods:**

We investigated whether early valproic acid treatment would prevent behavioral, cellular and gene expression abnormalities in *Disc1*-L100P mutants.

**Results:**

Valproic acid prevented hyperactivity and deficits in prepulse inhibition and latent inhibition in *Disc1*-L100P mice. Genome-wide transcription profiling identified *Lcn2* (*lipocalin2*) transcripts as being elevated by the *Disc1* mutation and corrected by valproate. *Disc1*-L100P mice also had increased glial cell numbers in the subventricular zone, which was normalized by valproate. Genetic deletion of *Lcn2* normalized glial cell numbers and behavior in *Disc1*-L100P mutants.

**Conclusions:**

Pharmacological treatments are a feasible way of preventing abnormal behaviour in a genetic model of schizophrenia. *Lcn2* is a potential novel drug target for early intervention in schizophrenia.

## Introduction

Schizophrenia is a neurodevelopmental disorder in which genetic and environmental factors disturb brain development, but the symptoms typically do not emerge until early adulthood [1]. Disrupted-In-Schizophrenia-1(*DISC1*) is a risk gene for schizophrenia and other mental illnesses, [2] encoding a scaffold protein [3] that is critical for adult neurogenesis [4], [5], neuronal migration, dendrite maturation and synaptogenesis [6], [7]. Better understanding of the molecular mechanisms by which *DISC1* variation alters neurodevelopment could lead to new treatment targets for neuropsychiatric illness.

There are a number of mouse models with *DISC1* mutations or altered DISC1 expression [2], demonstrating that the neurobiological and behavioral effects of DISC1 perturbation are time dependent [8], [9]. For example, transient expression of the DISC1 C-terminus on postnatal day 7 had effects not seen when expression was induced in adulthood [8]. The timing of expression of truncated DISC1 also has distinct behavioral effects [9]. Although behavioral impairments in various mutant *DISC1* mouse models have been observed in adult mice [10], the behavior of these mice as young adults has not yet been investigated.

We previously characterized *Disc1*-L100P mice generated by ENU mutagenesis [11]. *Disc1*-L100P mutants have disrupted pre-pulse inhibition (PPI), latent inhibition (LI), and working memory, in addition to hyperactivity. These schizophrenia endophenotypes [12], were corrected by antipsychotic treatment [11], [13]. *Disc1*-L100P mice are more sensitive to amphetamine, and have more D_2_ receptors in the striatum [13]. The *Disc1*-L100P mutation also reduced interactions between DISC1 and phosphodiesterase 4B (PDE4B) [11], and with glycogen synthase kinase-3 (GSK-3) [14]. Both rolipram (a PDE4B inhibitor) and TDZD-8 (a GSK-3 inhibitor) normalized behavior in *Disc1*-L100P mutants [11], [14]. Furthermore, the *Disc1*-L100P mutation impaired cortical development and cellular architecture [7], and affects expression of synaptic modulators neurexin 1 and 3 (Nrxn1/3) at critical developmental periods [15].

Because the *Disc1*-L100P mouse has many features consistent with schizophrenia, we sought to determine whether there was also an early adult onset of behavioral abnormalities. We found the behavior of 8 week-old mice to be normal [11], [13], [14], so we hypothesized that early intervention could rectify neurodevelopment and prevent abnormal behaviors emerging later in adulthood (12 weeks of age). Valproic acid (2-propylpentanoic acid) [16], given to *Disc1*-L100P mutant mice in early adulthood, prevented the emergence of schizophrenia-related behaviors later on. We profiled gene transcription in brain and identified higher *Lcn2 (Lipocalin2)* transcript levels in *Disc1*-L100P mice that were normalized by valproic acid. *Lcn2* is co-expressed with glial fibrillary acidic protein (GFAP) in brain, and the increased number of glial cells in *Disc1*-L100P mutants was corrected by valproic acid. Genetic ablation of *Lcn2* [17] normalized both behavior and glial cell numbers in *Disc1*-L100P mutants. Our work demonstrates that pharmacological intervention can prevent the onset of schizophrenia-related behaviors and suggests *Lcn2* as a novel drug target for preventive treatment of schizophrenia.

**Figure 1:**
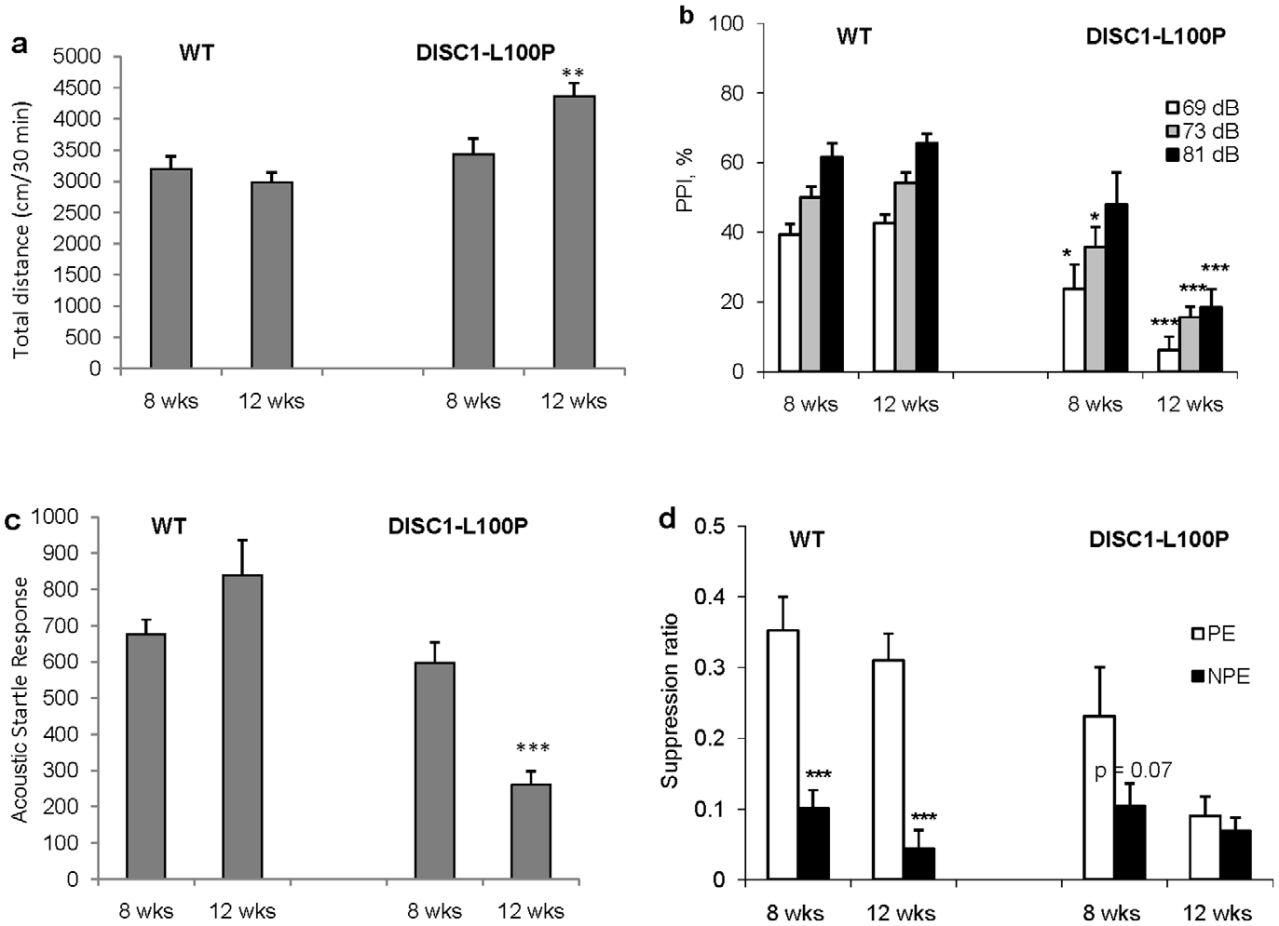
*Disc1*-L100P mutant mice show schizophrenia-related behavior at 12 but not 8 weeks of age. (**a**) 12 week-old *Disc1*-L100P mutant mice were hyperactive (n = 7−19 per group) as measured by total distance traveled during 30 minutes in the open field [F_1,22_ = 4.5, p<0.05 effect of genotype; F_1,22_ = 17.1, p<0.001 effect of age; F_1,22_ = 9.4, p<0.01 gene × age interaction]. (**b**) 12 week-old *Disc1*-L100P mutant mice have impaired PPI (n = 7−10 per group) [F_1,49_ = 58.8, p<0.001 effect of genotype; F_1,49_ = 4.97, p≤0.05 age; F_2,98_ = 150.2, p<0.001 prepulse intensity; F_1,49_ = 9.3, p<0.01 gene × age interaction]. (**c**) 12 week-old *Disc1*-L100P mutant mice have reduced Acoustic Startle Response (ASR) (n = 7−10 per group) [F_1,49_ = 19.9, p<0.001 genotype; F_1,49_ = 8.6, p<0.01 gene × age interactions]. *p<0.05, **p<0.01, ***p<0.001 in comparison with WT mice within each age group. (**d**) 12 week-old *Disc1*-L100P mutant mice have disrupted LI (n = 6−8 per group) [F_1,47_ = 19.1, p<0.001 genotype; F_1,47_ = 15.4, p<0.001 age; F_1,47_ = 49.1 p<0.001 effect of pre-exposure; F_1,47_ = 4.5, p<0.05 gene × age × pre-exposure interactions]. The eight experimental groups did not differ in A periods (all p>0.05, overall mean A period = 6.8 sec). ***p<0.001 non-pre-exposed (NPE) in comparison with pre-exposed (PE) animals to the conditioned stimulus (CS) within each genotype and age group. See also **Supplementary Table S1**.

## Materials and Methods

### Ethics Statement

All animal procedures were approved by the Toronto Centre for Phenogenomics (TCP) Animal Care Committee (AUP number 12-0025a-H) and followed the requirements of the Province of Ontario Animals for Research Act and the Canadian Council on Animal Care.

### Animals

*Disc1*-L100P homozygous mutants were generated as previously described [11], and backcrossed with C57BL/6 mice for 10–14 generations. Experiments were performed with 8, 12 and 15 week-old animals in sex-balanced groups. *Lcn2*-KO mice were backcrossed with C57BL/6J for 10 generations [18] and bred with *Disc1*-L100P homozygous mice to obtain WT, heterozygous and homozygous *Disc1*-L100P mice having two, one or no copies of *Lcn2*. Groups of 3–5 same-sex littermates were housed in filtered polycarbonate cages at 21±1°C, lights on 0700 h-1900 h and humidity at 50–60%. Animals were fed sterile Purina mouse chow and water *ad libitum*, except in the latent inhibition experiments. Behavioral experiments were conducted blind to genotype and drug treatment.

**Figure 2:**
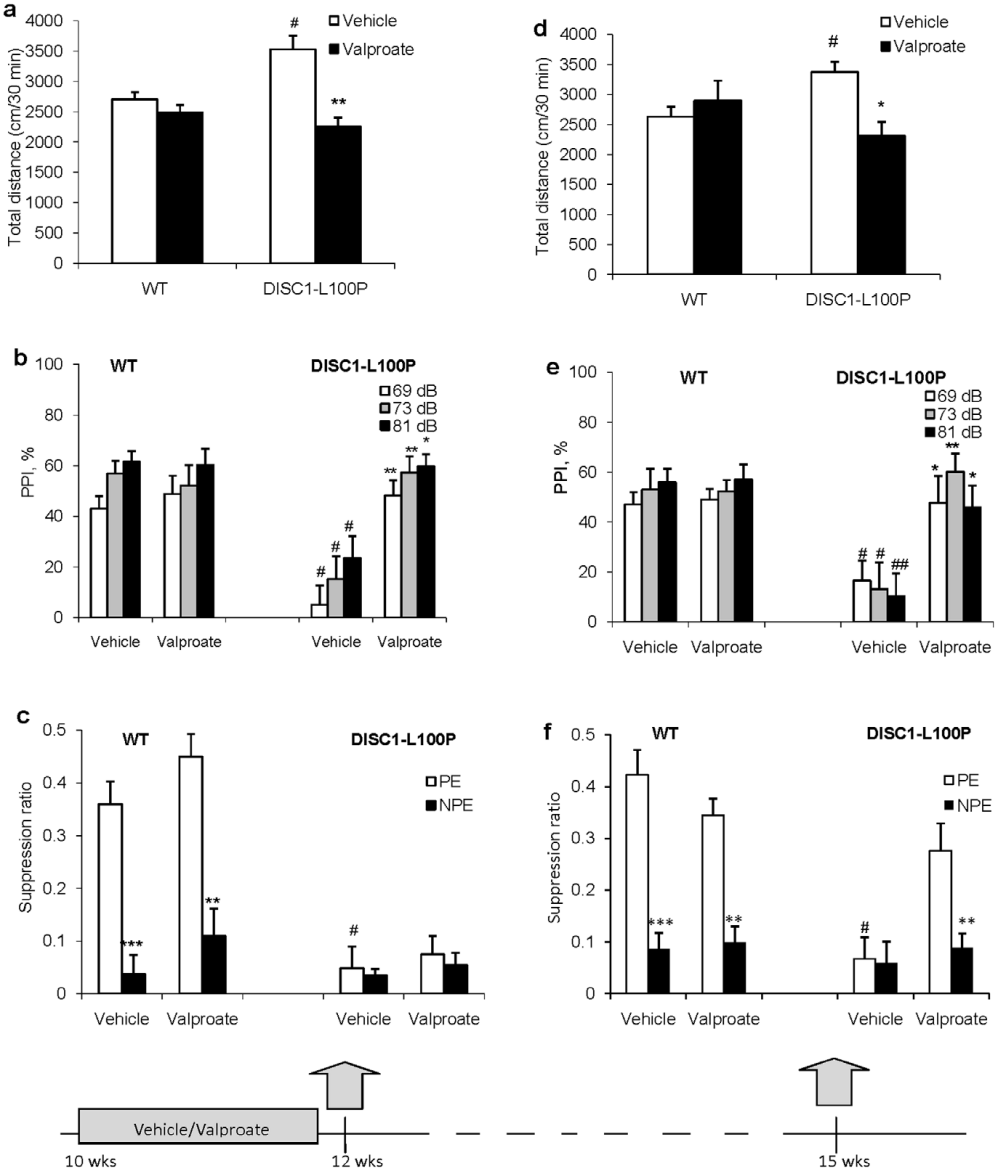
Chronic treatment with valproic acid between 10 and 12 weeks of age prevented the emergence of hyperactivity and PPI deficits in *Disc1*-L100P mutants and had a delayed effect on LI. (**a**) Valproic acid prevented hyperactivity in *Disc1*-L100P mice, but had no effect on locomotion of WT mice (n = 8−15 per group) [F_1,41_ = 8.4, p<0.001 genotype; F_1,41_ = 12.5, p<0.001 drug treatment; F_1,41_ = 5.6, p<0.05 gene × drug interaction. (**b**) Valproic acid prevented PPI deficits in *Disc1*-L100P mice. ANOVA showed a significant main effect of genotype [F_1,41_ = 7.6, p<0.01 genotype; F_1,41_ = 5.5, p<0.05 drug treatment; F_2,82_ = 4.4, p<0.05 pre-pulse intensity; F_1,41_ = 9.6, p<0.01 gene × drug interaction], #p<0.001 in comparison with vehicle-treated WT mice; *p<0.05, **p<0.01, ***p<0.001 in comparison with vehicle-treated *Disc1*-L100P. (**c**) Valproic acid did not prevent disrupted LI in *Disc1*-L100P mice [F_1,43_ = 51.1, p<0.001 genotype; F_1,43_ = 43.0 p<0.001 pre-exposure, and F_1,43_ = 33.5, p<0.001 genotype × pre-exposure]. The eight experimental groups did not differ in A periods (all p>0.05, overall mean A period  = 7.2 sec). #p<0.001 non-pre-exposed (NPE) in comparison with pre-exposed (PE) vehicle-treated WT to the conditioned stimulus (CS); **p<0.01; ***p<0.001 NPE in comparison with PE animals within each genotype and drug treatment. (**d-g**) Valproic acid had lasting effects on behavior in *Disc1*-L100P mutants 3 weeks after the last dose. (**d**) Valproic acid-treated *Disc1*-L100P and WT mice had similar locomotion, whereas vehicle-treated *Disc1*-L100P mutants were hyperactive in comparison with vehicle-treated WT mice; *p<0.05 vs. vehicle-treated WT mice; *p<0.05 vs. vehicle-treated *Disc1*-L100P mice. (**e**) Valproic acid prevention of PPI deficits in *Disc1*-L100P mice persisted 3 weeks after drug treatment ended [F_1,40_ = 5.7, p<0.05] as compared with vehicle-treated *Disc1*-L100P mutants (p<0.05 at 69 dB and 81 dB; p<0.01 at 73 dB). In contrast, vehicle-treated *Disc1*-L100P mice showed PPI deficits [F_1,40_ = 5.54, p<0.05] in comparison with vehicle-treated WT animals at all three pre-pulses (p<0.05 at 69 and 73 dB, p<0.01 at 81 dB). #p<0.05, ##p<0.01 in comparison with vehicle-treated WT mice; *p<0.05; **p<0.01 in comparison with vehicle-treated *Disc1*-L100P mice. (**f**) *Disc1*-L100P mice treated with valproic acid developed LI three weeks after treatment had stopped. Valproic acid-treated *Disc1*-L100P mice showed LI (p<0.01), similar to vehicle- and valproic acid-treated WT mice (p<0.001 and p<0.01, respectively), but no LI in vehicle-treated *Disc1*-L100P mice (p>0.05). #p<0.001 in comparison with vehicle-treated WT mice; **p<0.01; ***p<0.001 NPE in comparison with PE animals within each genotype and drug treatment. ANOVA detected a significant effect of genotype [F_1,43_ = 12.8, p<0.001], drug treatment [F_1,43_ = 15.2 p<0.001], pre-exposure [F_1,43_ = 47.9 p<0.001] and gene × drug × pre-exposure interactions [F_1,43_ = 6.1, p<0.05]. There was no difference in A periods among the eight experimental groups (all p>0.05, overall mean A period = 8.1 sec).

### Behavioral Assays

#### Locomotor activity

Monitored for 30 minutes in a directly illuminated (600 Lux) clear Perspex chamber (42 cm×42 cm×30 cm; Accuscan Instruments Inc, Ohio, USA) by interruptions of 16 horizontal and 16 vertical infrared beam sensors 2.5 cm apart.

#### Pre-pulse Inhibition (PPI) of Acoustic Startle Response (ASR)

Measured as previously described [11]. Five types of trials were used: (i) Pulse alone - single white noise burst (120 dB 40 ms); (ii) Prepulse+pulse (PP69P, PP73P, PP81P) – noise prepulse (20 ms at 69, 73, or 81 dB) followed by startle pulse (120 dB 40 ms) 100 ms after the prepulse onset; (iii) No-stimulus - background noise only (65 dB). Sessions were structured as follows: (i) 15-minute acclimation at background noise level; (ii) five Pulse trials; (iii) ten blocks each containing all five trials (Pulse, PP69P, PP73P, PP81P, No-stimulus) in pseudorandom order; (iv) five Pulse trials. The force intensity for each trial was recorded as the startle level. The percentage PPI induced by each prepulse intensity was calculated as [1-(startle amplitude on prepulse trial)/(startle amplitude on pulse alone)]*100%.

#### Latent Inhibition (LI)

Assessed as previously described [11]. Mice were trained to drink in the experimental chamber for 5 days, 15 minutes per day. The LI procedure consisted of *Pre-exposure*, *Conditioning, Lick Retraining* and *Test* sessions. *Pre-exposure:* The pre-exposed (PE) mice received 40 white noise presentations (60 s inter-stimulus interval). The non-pre-exposed (NPE) mice were confined to the chamber for the same time without receiving the stimuli. *Conditioning:* All mice received fear conditioning to the noise stimulus. Two noise-shock pairings were used (10 s 85 dB white noise; 1 s 0.37 mA shock). *Test:* The noise was activated between licks 75–101. The following times were recorded: time to first lick, time to complete licks 50–75 (before noise onset: A period) and time to complete licks 76–101 (after noise onset: B period). The suppression ratio was calculated as A/(A+B).

### Drug Administration Schedule

Valproic acid sodium salt (Sigma-Aldrich, Canada) in 0.9% NaCl was injected intraperitoneally (i.p) at 200 mg/kg, twice daily for 14 days [19]. Behavioral testing started 20 hours after the last injection (LI sessions were between injections), using independent cohorts for each test and for microarrays, Western blotting, and immunohistochemistry. Mice for tissue analysis were killed 20 hours after the last valproate injection, either by cervical dislocation, or anaesthetized with pentobarbital.

**Figure 3:**
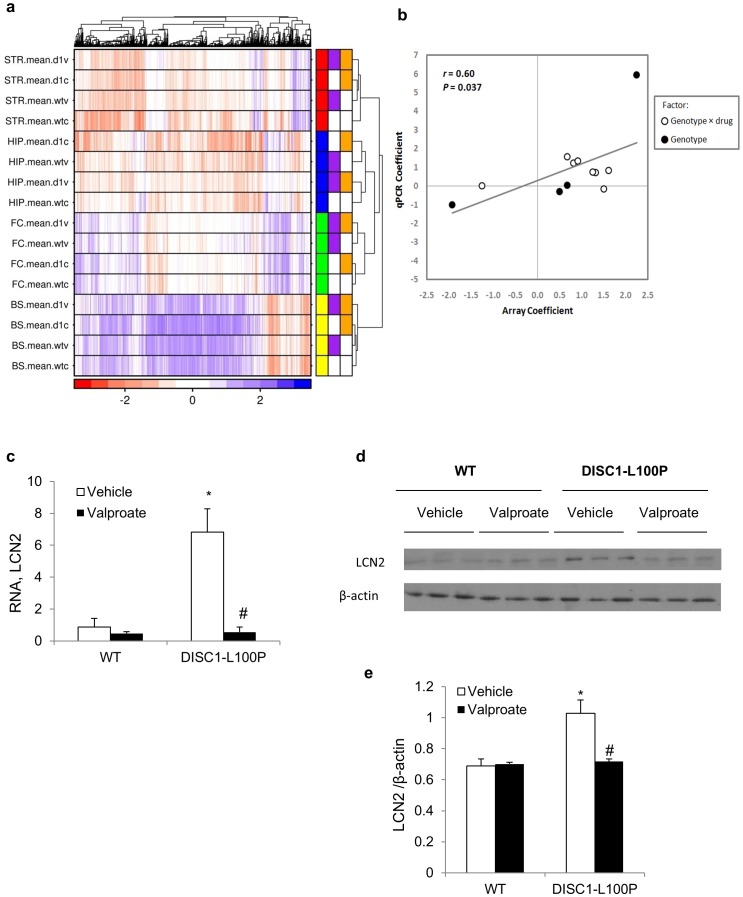
Gene expression changes induced by the *Disc1*-L100P mutation and by treatment with valproic acid. (**a**) Heatmap showing expression microarray results. Columns indicate individual genes, rows individual tuples (brain region, genotype, treatment). The color legend along the bottom indicates the scaled signal intensity, with blue indicating high abundance. The colored boxes at right indicate, from left to right: the brain region (red - striatum, blue - hippocampus, green - frontal cortex, yellow - brain stem), treatment (purple - valproic acid, white - vehicle), and genotype (orange - *Disc1*-L100P, white - WT). BS - brain stem, FC - frontal cortex, HIP – hippocampus, STR - striatum. d1v - valproate-treated *Disc1*-L100P mutants; d1c - vehicle-treated *Disc1*-L100P mutants; wtv - valproate-treated WT; wtc - vehicle-treated WT. (**b**) Validation of array results: *Arc*, *Egr2*, *Dusp1*, *Purb*, *Slc40a1*, *Adar*, *Mrpl39*, *Slc6a12*, *Lcn2*, *Igf1*, *EiF4ebp2* and *Cyr61* transcripts were analyzed by RT-qPCR. Coefficients refer to either the genotype-drug interaction term or the genotype term in ANOVA analyses. For microarrays, this analysis was performed using the fold-change magnitude in log 2-space, for RT-qPCR, RNE values (log 2-based fold changes in mRNA quantity, normalized to *Gapdh* and *β-actin* levels). We obtained an overall validation rate of 0.60 (P = 0.037). *r*, Pearson correlation factor. (**c-e**) Elevated *Lcn2* (*Lipocalin 2*) expression in *Disc1*-L100P mutant mice normalized by valproic acid treatment. (**c**) *Lcn2* mRNA levels assessed by qRT-PCR (n = 5−6 per group). RNE values are relative to GAPDH and β-actin. ANOVA detected a significant effect of genotype [F_1,15_ = 18.4, p<0.001], drug [F_1,15_ = 20.6, p<0.001] and gene × drug interaction [F_1,15_ = 18.2, p<0.001] on *Lcn2* expression. (**d**) Protein levels in representative Western blots are shown, probed with an antibodies against LCN2 and β-actin as a loading control in extracts of brain stem isolated from vehicle- and valproate-treated *Disc1*-L100P and WT mice (n = 6−7 per group). (**e**) Densitometric analysis to quantify the relative intensity of LCN2-immunoreactive bands relative to β-actin. ANOVA found a significant effect of genotype [F_1,20_ = 31.4, p<0.001], drug [F_1,20_ = 21.9, p<0.001] and their interaction [F_1,20_ = 25.9, p<0.001] on LCN2 protein levels. *p<0.05 in comparison with vehicle-treated WT; #p<0.05 in comparison with vehicle-treated *Disc1*-L100P mutants. See also **Supplementary Tables S2–6**.

### Preparation of Total RNA

Gross brain dissections were performed to obtain brain stem (BS), frontal cortex (FC), hippocampus (HIP), and striatum (STR) samples, from which total RNA was extracted as described previously [20]. Samples were mechanically homogenized and lysed using Trizol® Reagent (Invitrogen, Carlsbad, CA). RNA was precipitated with 70% ethanol, and purified and treated with DNAase using the Absolutely RNA Miniprep® kit (Stratagene, La Jolla, CA).

### Target Preparation and Microarray Hybridization

Microarray hybridization and scanning were done at the Génome Québec Innovation Centre (Montreal, QC). RNA samples were quantified using the NanoDrop ND-1000 UV-Vis Spectrophotometer (Thermo Fisher Scientific, Waltham, MA), and evaluated using the Agilent Bioanalyzer 2100 (*Agilent* Technologies, Santa Clara, CA). Complementary RNA (cRNA) was amplified and biotinylated using the Illumina TotalPrep Amplification Kit for 3–6 replicates within each treatment-genotype combination and hybridized to MouseRef-8 v2.0 Expression BeadChips (>25,000 probes; Illumina, San Diego, CA). BeadScan software (version 3, 2006) was used to identify bead positions and to extract raw data.

**Table 1:** The number of unique Illumina beads identified as differentially expressed in each of the three conditions and four brain regions.

Brain Region	Valproate	DISC1-L100P	Interaction
Brain stem	0	13	2
Frontal cortex	0	0	2
Striatum	2	0	0
Hippocampus	3	61	30

### Microarray Analysis

Each brain region was analyzed independently and identically. A two-factor, two-level design was used, with the factors being drug treatment and genotype. Raw array data were loaded into the beadarray package (v1.10.0) of BioConductor [21]. Following BASH analysis to remove spatial artifacts [22], data were pre-processed using Edwards background correction [23] and normalized using variance-stabilization (vsn package of BioConductor v3.8.0) [24]. A two-level, two-factor general linear model was then fit to the pre-processed expression values from each bead type using the limma package (v2.16.3) of BioConductor, followed by an empirical Bayes moderation of the standard error and a false-discovery rate (FDR) adjustment for multiple-testing. Genes were selected at a 10% FDR threshold in any contrast. Both normalized and pre-processed array data are available in GEO (GSE17735). Data were visualized using unsupervised machine-learning [25]. The mean normalized signal intensities for each (tissue, genotype, treatment) tuple were collated into a single matrix, with rows as genes and columns as conditions. Rows were selected based on sequential variance (0.1, 0.25, 0.5, 1.0) or signal intensity (7,8,9,10,11,12) thresholds. This matrix was mean-centered and root-mean-square-scaled, and then subjected to agglomerative hierarchical clustering using complete linkage with Pearson’s correlation as a distance metric. Heatmaps were generated in the R statistical environment (v2.8.1) using the lattice (v0.17–20) and lattice Extra (v0.5-4) packages. Functional analysis was performed using the NIAID’s DAVID resource (v2008).

**Figure 4:**
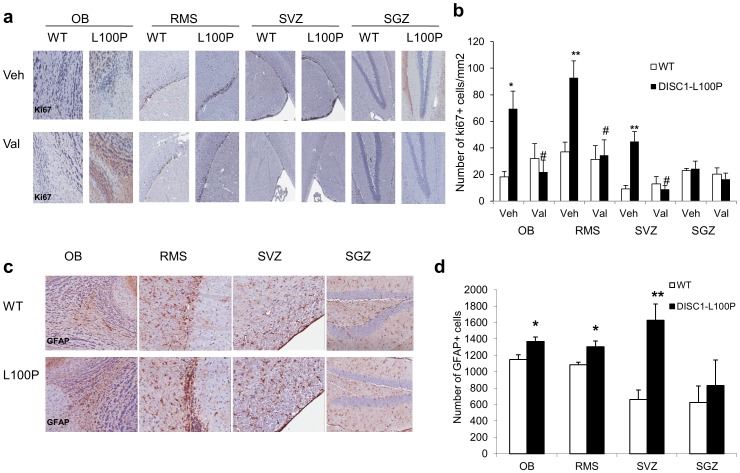
Increased GFAP+ cell proliferation in *Disc1*-L100P mutants normalized by valproic acid. (**a**) Representative examples of brain sections from olfactory bulb (OB), rostral migratory stream (RMS), subventricular zone (SVZ), and subgranular zone (SGZ) of the hippocampus of vehicle-treated (top) or valproate-treated (bottom) WT and *Disc1*-L100P mice, immunostained for ki67 (dark brown). All images were taken at 10× magnification. (**b**) Quantitative analysis of the number of ki67^+^ nuclei in OB, RMS, SVZ and SGZ in all gene and drug conditions. **OB:** There was a significant effect of genotype [F_1,62_ = 18.23; p<0.01]; drug [F_1,62_ = 18.9; p<0.01] and gene × drug interactions [F_1,62_ = 19,14; p<0.01]. **RMS:** There was an effect of genotype [F_1,62_ = 17.38, p<0.01]; drug [F_1,62_ = 28.47, p<0.001] and their interaction [F_1,62_ = 15.71, p<0.05]. **SVZ:** There was a significant effect of genotype [F_1,62_ = 28.54, p<0.01]; drug [F_1,62_ = 16.28; p<0.05] and their interactions [F_1,62_ = 16.54, p<0.01]. ANOVA did not detect an effect of genotype and/or drug treatment on ki67^+^ cells in **SGZ** (all p>0.05). The number of ki67^+^ cells were expressed as the number of cells per 1 mm^2^ using Aperio Image Scope. *p<0.05; **p<0.01 in comparison with vehicle-treated WT mice; #p<0.01 in comparison with vehicle-treated *Disc1*-L100P mice; Veh – vehicle; Val – valproate; n = 4 sections per animal from 4–6 mice; (**c**) Increased GFAP^+^ cells in OB, RMS and SVZ but not in SGZ of vehicle-treated *Disc1*-L100P mice as compared to WT, assessed by GFAP immunostaining (brown). All images were taken at 20× magnification. (**d**) Quantitative analysis of the number of GFAP^+^ nuclei in OB, RMS, SVZ and SGZ. ANOVA detected a significant effect of genotype in **OB** [F_1,30_ = 7.71; p<0.01], **RMS** [F_1,30_ = 8.18, p<0.01], **SVZ** [F_1,30_ = 8.81, p<0.05] but not in **SGZ** [F_1,30_ = 0.39, p>0.05]. The number of GFAP^+^ cells were expressed as the number of cells per 1 mm^2^ using Aperio Image Scope. *p<0.05; **p<0.01 in comparison with WT mice. n = 4 sections per animal from 4–6 mice. See also **Supplementary Table S7**.

### Verification of Microarray by Quantitative RT-PCR

The mRNA levels of *Adar*, *Egr2*, *Slc40a1*, *Mrpl39*, *Slc6a12*, *Lcn2*, *Arc*, *Dusp1*, *Purb*, *Cyr61*, *Igf1* and *eiF4ebp2* were measured using quantitative RT-PCR (qRT-PCR). cDNA was prepared using the Omniscript Reverse Transcription kit (Qiagen, Valencia, CA), and qRT-PCR was performed in quadruplicate using a reaction volume of 50 µL on the ABI PRISM 7500 (Applied Biosystems Inc., Foster City, CA). The qRT-PCR cycle consisted of activation of AmpErase UNG (50°C, 2 min), Taq activation (95°C, 10 min), 40 cycles of denaturation (95°C, 15 sec), and elongation (60°C, 1 min) during which fluorescence was measured. Gene expression was quantified using *Gapdh* and *β-actin* as endogenous controls for normalization. All assays were from ABI. Correlation between microarray data (fold-change in log 2-space) and qRT-PCR data (fold-change in log 2-space, normalized to *Gapdh* and *β-actin*; expressed as relative normalized expression, RNE, values) was analyzed in R (v2.9.1).

**Figure 5:**
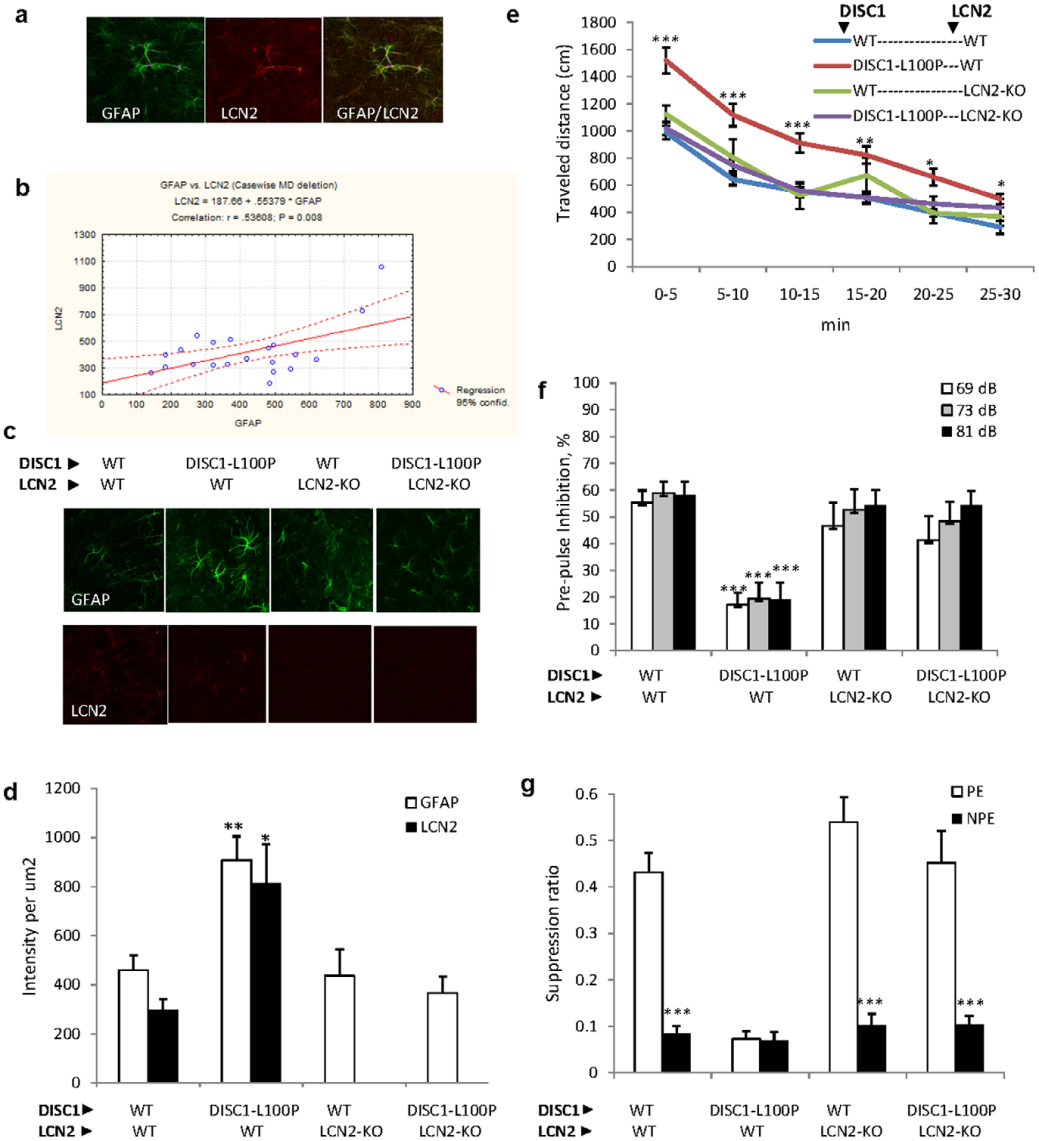
LCN2 is co-expressed with GFAP in the brain and levels are associated with behavior in *Disc1*-L100P mice. (**a**) Co-expression of LCN2 with GFAP in the mouse brain shown in confocal microscopic images of cortical slices stained with LCN2 and GFAP antibodies (20× magnification). (**b**) Correlation of LCN2 and GFAP levels in different brain areas, including olfactory bulbs, rostral migratory stream, subventricular zone (SVZ), subgranular zone of hippocampus and cortex (n = 21). Levels of GFAP^+^ and LCN2^+^ staining were expressed as the intensity per 1 um^2^ using a computerized image analyzer (EZ-C1, gold v3.9, Nikon Corporation). (**c-d**) Genetic inactivation of *Lcn2* corrected the increased number of GFAP^+^ cells in SVZ of *Disc1*-L100P mutant mice. (**c**). Confocal images of slices of the SVZ probed with LCN2 and GFAP in 4 genotypes: wild-type (WT); *Disc1*-L100P, *Lcn2*-KO and *Disc1*-L100P × *Lcn2*-KO (n = 4 slices per mouse; 3–5 mice per genotype; 20× magnification). (**d**)**.** Quantitative analysis of LCN2 and GFAP expression in SVZ in mice of 4 genotypes. *p<0.05; **p<0.01 in comparison with WT; unpaired *t-test*. (**e-g**)**.** Genetic inhibition of *Lcn2* corrected behavior in *Disc1*-L100P mutant mice, including hyperactivity, pre-pulse inhibition (PPI) and latent inhibition (LI). (**e**) Locomotor activity in the open field in WT or *Disc1*-L100P mice carrying both *Lcn2* alleles (WT) or missing both *Lcn2* alleles (LCN2-KO). ANOVA with repeated measures found a main effect of genotype [F_3,43_ = 11.4, p<0.001], testing interval [F_5,215_ = 137.4, p<0.001] and genotype × testing interval interaction [F_15,215_ = 3.2, p<0.001]. *Disc1*-L100P mutants were hyperactive at all tested intervals (p<0.001 during the first 15 minutes, p<0.01 at 15–20 minutes and p<0.05 at 20–30 minutes), whereas genetic ablation of *Lcn2* rendered their activity the same as WT mice. n = 6−17 mice per genotype. *p<0.05; **p<0.01; ***p<0.001 in comparison with WT mice. (**f**) PPI deficit in *Disc1*-L100P mutants was normalized at all three pre-pulses by genetic ablation of *Lcn2*. ANOVA with repeated measures found a main effect of genotype [F_3,31_ = 14.1, p<0.001], and pre-pulse intensities [F_2,62_ = 6.6, p<0.05]. n = 6−11 mice per genotype. ***p<0.001 in comparison with WT mice (**g**) Deletion of *Lcn2* in *Disc1*-L100P mutants also restored LI. ANOVA detected a significant effect of genotype [F_3,47_ = 13.5; p<0.001], pre-exposure [F_1,47_ = 62.4; p<0.001] and their interaction [F_3,47_ = 9.9, p<0.001]. n = 6−8 per experimental group. ***p<0.001 non-pre-exposed (NPE) in comparison with pre-exposed (PE) animals to the conditioned stimulus (CS) within each genotype.

### Western Blotting

Tissues were homogenized in RIPA buffer (Sigma-Aldrich). After centrifugation at 10,000 *g* at 4°C for 20 min, the supernatant was extracted and protein concentrations were measured by Bradford assay. Aliquots of protein extract (20–40 µg) were boiled with Laemmli sample buffer, separated by 10–15% SDS-PAGE, and transferred to PVDF membranes (Life Sciences). Blots were immunostained overnight at 4°C with antibodies from Santa Cruz Biotechnology: rat anti-LCN2 monoclonal (1:1000), rabbit anti-Dusp1 polyclonal (1:1000), goat anti-Cyr61 polyclonal (1:1000), and rabbit β-actin polyclonal (1:2000). Immune complexes were detected using appropriate peroxidase-conjugated secondary antibodies and a chemiluminescent reagent (ThermoScientific).

**Figure 6:**
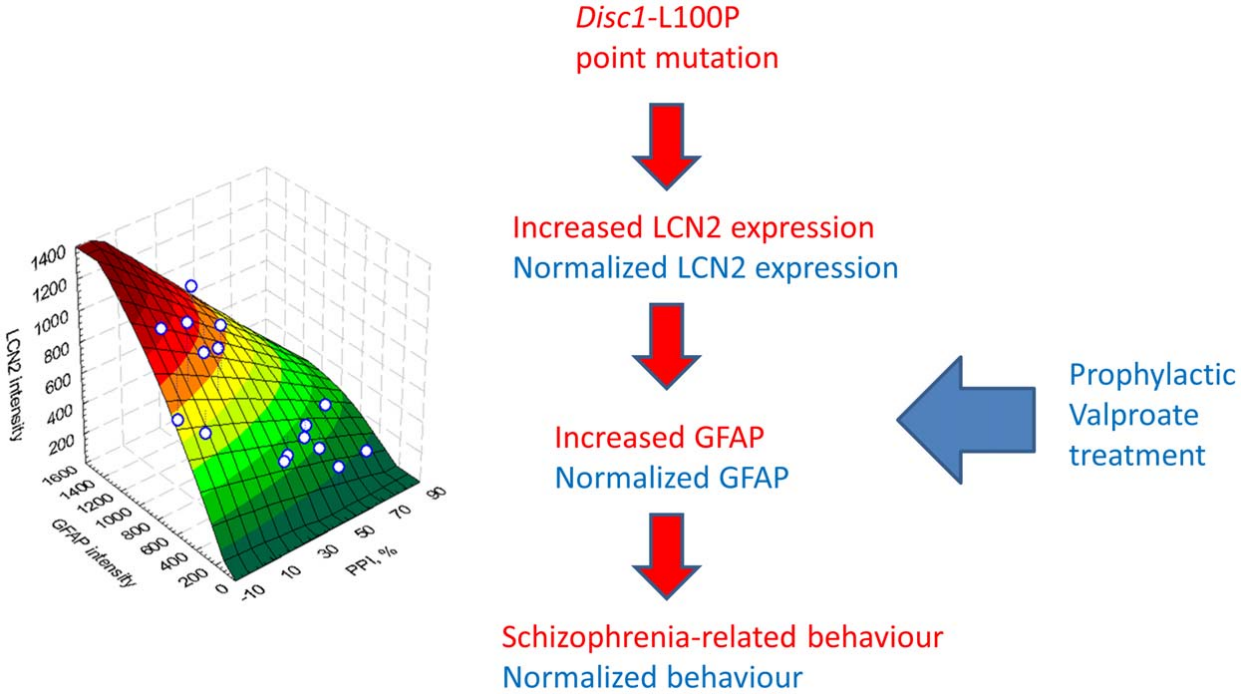
Summary of results. Lcn2 levels correlate with GFAP in subventricular zone (SVZ) and these are in turn associated with abnormal PPI, an endophenotype for schizophrenia in *Disc1-L100P* mice. The 3D quadratic surface graph illustrates surfaces fitted by a smoothing technique to the average PPI, GFAP and Lcn2 intensity in SVZ data. The color spectrum (from green to brown) represents the GFAP and Lcn2 intensity data (from low to high, respectively). Pearson correlation coefficients are: r = −0.73; p<0.001 – for PPI and Lcn2; r = −0.56; p<0.05 – for PPI and GFAP and r = 0.77, p<0.001– for Lcn2 and GFAP (N = 16).

### Immunohistochemistry

After pentobarbital anesthesia (120 mg/kg), mice were perfused transcardially with 4% periodate-lysine-paraformaldehyde at 4°C. Paraffin-embedded brain was cut into 5 mm thick sagittal sections, de-paraffined with xylene and rehydrated with ethanol. After blocking with Dako, the sections were incubated with primary antibodies overnight (rabbit anti-ki67, Lab Vision, 1:200), anti-neuronal nuclear protein (anti-NeuN, Chemicon, 1:200), or anti-glial fibrillary acidic protein (anti-GFAP, COVANCE, 1:200), followed by incubation with secondary antibody (goat anti-rabbit IgG, Vector Labs, 1:200) and tertiary antibody (ABC, Vector labs, 1:50). DAB color development was followed by scanning (Aperio Scanner). Apoptosis was assessed by TUNEL assay (Roche Applied Science) following the manufacturer’s protocol. Cells were counted using Aperio ImageScope software. At least four animals per condition (genotype, drug) were used, and a total of four sections per animal were analyzed.

### Confocal Microscopy

After pentobarbital anesthesia and perfusion as above, whole brain was immersed in 4% PFA for 24 hours, and 100 µm sagittal slices were cut on a vibratome. Next, slices were immersed in 4% BSA, 0.1% Triton-X 100, PBS solution for 15 minutes. Primary antibodies (rabbit anti-LCN2 polyclonal, Santa Cruz Biotechnology, 1:100) and mouse anti-GFAP (1:250) were incubated with free-floating slices for 48 hours at 4°C. Slices were washed 3×10 minutes in blocking-permeabilizing solution and incubated with secondary antibodies (rabbit LCN2-CY5, 1:200 or mouse GFAP-CY2, 1:200) for 2 hours at 20°C. Following incubation, slices were washed again (3×10 minutes) and mounted on glass slides. For quantitative studies 3–5 mice per genotype and 4 slices per mouse were examined (Zeiss LSM 510 confocal microscope). All images were acquired with fixed exposure settings and analysed using Nikon EZ-C1 FreeViewer v3.9.

### Statistical Analysis

The effects of the *Disc1*-L100P mutation, age, valproate treatment and *Lcn2* deletion on mouse behaviors, cell counts, and mRNA or protein levels were evaluated by ANOVA or unpaired *t-test*. Because there were no significant sex effects, data for both sexes were combined. Significant main effects or interactions were followed by the Fisher’s least significance difference (LSD) post-hoc test with significance set at p<0.05.

## Results

### *Disc1*-L100P Mice Behave Abnormally at 12 but not at 8 Weeks of Age

We assessed behavior of *Disc1*-L100P mutants at 8 weeks of age, when PPI and LI normally develops [26], [27], [28], and later in adulthood (12 weeks of age). We focused on behaviors relevant to schizophrenia: hyperactivity, PPI, and LI [29]. *Disc1*-L100P mutants were hyperactive at 12 but not 8 weeks of age compared to WT mice (Figure 1a). A mild PPI deficit in 8 week-old *Disc1*-L100P mice became more pronounced at 12 weeks (Figure 1b). *Disc1*-L100P mice had normal Acoustic Startle Response (ASR) at 8 weeks of age, which decreased significantly by 12 weeks of age (Figure 1c). Similarly, young adult *Disc1*-L100P mutant mice showed LI, but had disrupted LI at 12 weeks of age, whereas WT mice showed robust LI at both ages (Figure 1d).

### Valproic Acid Prevented Schizophrenia-related Behavior in *Disc1*-L100P Mice

Valproic acid treatment between 10 and 12 weeks of age prevented hyperactivity, PPI deficits and ameliorated ASR, but did not immediately correct LI deficits in *Disc1*-L100P mice (Figure 2a–c and **Table S1**). All behaviours in valproic acid-treated *Disc1*-L100P mice were comparable with WT mice, three weeks after the last dose of valproic acid (Figure 2d–f). LI actually improved in the three weeks after valproic acid treatment stopped. These data show that valproic acid treatment had lasting effects on behavior, even after the medication had been discontinued.

### Transcriptomic Analysis

The *Disc1*-L100P mutation had the most pronounced effect on gene expression in the hippocampus (61 genes), with 13 genes showing altered expression in brain stem, but no changes in gene expression in striatum or frontal cortex (Table 1 and Figure 3a). Functional annotation of genes in the hippocampus revealed that most are involved in cell proliferation (23%) and cytoskeleton/cell shape (15%). Valproic acid significantly altered expression of 3 genes in hippocampus (*Hist1h1c, Hist1h2be and Clcn2)* and 2 genes in striatum (*Egr2* and *Fosb*). Among 61 genes altered by the *Disc1*-L100P mutation, valproate corrected the expression of 13 genes (21.3%) in hippocampus and *Lcn2* in the brain stem. Expression of 14 genes was changed only in valproate-treated Disc1*-L100P* mutants. The specific genes are listed in **Tables S2-S6**.

### Verification of Gene Expression by qRT-PCR

We used qRT-PCR to validate mRNA levels of genes for which: (1) the effect of *Disc1*-L100P mutation was accompanied by the opposite effect of valproic acid (genotype × drug interaction) on gene expression (*Arc*, *Purb*, *Egr2*, *Dusp1*, *Slc40a1*, *Mrpl39*, and *Igf1*), (2) *Disc1*-L100P mutation alone had a significant effect on expression (*Slc6a12*, *Adar*, *Lcn2* and *EiF4ebp2*), and (3) genotype × drug interaction had a significant effect on expression (*Cyr61*). Correlation analysis of microarray and qRT-PCR data validated 7/12 genes (*Egr2*, *Slc40a1*, *Arc*, *Dusp1*, *Cyr61*, *Lcn2* and *Slc6a12*). We obtained an overall validation rate of 0.6 (Figure 3b; p = 0.037). The strongest candidate transcript was *Lcn2* (Figure 3c), and Lcn2 protein levels were consistent with qRT-PCR (Figure 3d–e). In particular, there was more *Lcn2* brainstem mRNA in *Disc1*-L100P mutants (p<0.05) that was normalized by valproic acid.

### Valproic Acid Corrected Increased Cell Proliferation in *Disc1*-L100P Mutants

Given that DISC1 [4], [5], [6] and LCN2 [30] are both involved in cell proliferation and that cell proliferation was the largest functional category of altered transcripts (23%), we performed immunohistochemistry on the cell proliferation marker Ki67 in vehicle/valproic acid-treated *Disc1*-L100P and WT mice. As shown in Figure 4a–b, vehicle-treated *Disc1*-L100P mutants had more Ki67+ cells in olfactory bulbs (OB), rostral migratory stream (RMS), and subventricular zone (SVZ), but not sub-granular zone (SGZ) of hippocampus. These are the main areas for adult neurogenesis [31]. Valproic acid no effect on Ki67+ cell counts in WT mice.

### *Disc1*-L100P Mutation Increased Glia but not Neurons in SVZ

To identify the proliferating cells in *Disc1*-L100P mutants, we performed immunohistochemistry with antibodies against neuronal nuclear protein (NeuN) and glial fibrillary acidic protein (GFAP). As shown in Figure 2c–d, there was a significant increase in GFAP-positive cells in OB, RMS and SVZ in *Disc1*-L100P mutants compared to WT mice but not in the hippocampus. Most post-mortem studies in schizophrenia report no or minimal gliosis [32], but some find gliosis in periventricular structures [33]. There was no difference in the number of NeuN-positive cells in all brain areas examined between the genotypes. We performed TUNEL staining to assess cell death and found no differences between *Disc1*-L100P and WT littermates. We also assessed cell proliferation in young animals and did not find significant differences between genotypes in the brain regions studied (**Table S7**).

### Genetic Deletion of *Lcn2* Corrected Excess GFAP^+^ Cells and Normalized Behavior in *Disc1*-L100P Mutant Mice

We sought to assess the role of *Lcn2* in mediating excess glial cells and schizophrenia-related behavior by crossing mice that lack both alleles of *Lcn2* to either WT mice or *Disc1*-L100P mutants [11], [17]. LCN2 is an autocrine mediator of reactive astrocytosis [34], which could be related to glial proliferation. Immunostaining with LCN2 and GFAP antibodies revealed their co-expression in OB, RMS, SVZ, cortex, hippocampus, and brain stem (Figure 5a). The expression levels of the two genes were positively correlated (r = 0.53, p<0.01) (Figure 5b). *Disc1*-L100P mutation increased LCN2 and GFAP proteins in the SVZ (Figure 5c–d) as well as in OB, RMS but not in the subgranular zone (SGZ). Genetic inactivation of *Lcn2* reduced the excess GFAP^+^ cells in SVZ (Figure 5c–d), OB and RMS with no effect in SGZ in *Disc1*-L100P mutant mice (**Table S8**). Finally, we assessed effect of genetic inactivation of *Lcn2* on schizophrenia-related behaviors. Ablation of both copies of *Lcn2* normalized locomotion, PPI, LI and ASR abnormalities of *Disc1*-L100P mutants to the level of WT control mice (Figure 5e–g).

## Discussion

Our results show that: (1) abnormal behaviors in *Disc1*-L100P mutant mice are present at 12 weeks but not 8 weeks of age; (2) treatment with valproic acid prevented the emergence of abnormal behaviors in *Disc1*-L100P mutants; (3) 23% of transcripts altered in *Disc1*-L100P mice are implicated in cell proliferation, including *Lcn2*; (4) there were more glial cells in the SVZ, RMS and OB but not in SGZ of *Disc1*-L100P, which was corrected by valproic acid; (5) genetic inactivation of *Lcn2* corrected excess glial cells and schizophrenia-related behavior in *Disc1*-L100P mutants (Figure 6). We have identified a novel function for *Lcn2* as a regulator of glial proliferation in the SVZ and of behaviors relevant to schizophrenia.

An important question about the pathophysiology of schizophrenia is why the psychotic symptoms emerge in the third decade of life, despite high heritability and the presence of premorbid abnormalities in early childhood [35]. Longitudinal magnetic resonance imaging (MRI) studies reveal progressive structural brain changes prior to the first episode of psychosis [36]. However, it remains unknown whether these premorbid structural abnormalities can be prevented by prophylactic early intervention. Many schizophrenia patients have a prodromal period [1], [37] of social and functional decline [38], that provides an opportunity for early intervention.

pWe have chosen to investigate the potential for valproic acid to prevent the emergence of behavioral abnormalities in the *Disc1*-L100P mouse model for several reasons. First, valproic acid is already approved for use in teenagers as a mood stabilizer and anticonvulsant [16], [39]. 15 Second, schizophrenia is caused by multiple factors that interact at many biological levels [40], so improved treatment may have to target multiple mechanisms [41]. Valproic acid is a drug with many pharmacological actions [16], [42], [43], [44], [45] and targets, including GABA transaminase, voltage-gated sodium channels, glycogen synthase kinase (GSK)-3, and histone deacetylases (HDACs) [16]. Thus, it was used as a screen to test if early intervention in an animal model for schizophrenia is feasible. Finally, we chose not to test antipsychotic medications because clinical trials during the prodrome of schizophrenia showed limited benefit [46].

To our knowledge, this is the first report of delayed onset of schizophrenia-related behaviors in a genetic animal model of schizophrenia. However, adolescent onset of schizophrenia-related behaviors has also been observed with prenatal immune activation by polyinosinic–polycytidilic acid (poly I:C) [47], and risperidone pre-treatment can prevent brain and behavioral abnormalities if administered in adolescence [48]. The beneficial effects of valproic acid on *Disc1*-L100P mice are similar to its efficacy in other mouse models of schizophrenia [19], [49], [50]. Importantly, we found that chronic valproic acid treatment prevented schizophrenia-related behaviors for three weeks after the last dose. Thus, we argue that valproic acid treatment may have altered brain development, rather than just having acute effects, which represents a new paradigm for preventive treatments [51].

Genome-wide expression profiling identified large effects of the *Disc1*-L100P mutation in the hippocampus, an area with high DISC1 expression [52]; smaller but still significant effects were also seen in the brain stem, consistent with a wide-ranging DISC1 functional network [53]. Valproic acid alone had minimal effects on gene expression in hippocampus and striatum, consistent with the limited behavioral effect on WT animals. As expected, valproic acid affected expression of transcription factors (*Egr2* and *Fosb*), genes involved in epigenetic regulation (*Hist1h1c* and *Hist1h2be*) and neuronal excitability (*Clcn2*) [16]. Valproate had broad transcriptional effects in DISC1 mutants, correcting 21.3% of altered gene expression in the hippocampus. Moreover, we detected convergent effects of both *Disc1*-L100P and valproate on expression of 14 genes, of which five are independently associated with schizophrenia (**Tables S2–6**).

Glial cells are important in synaptic function, maturation and elimination, and are thus a potential therapeutic target for brain disorders [54]. Here, we describe for the first time, an association of schizophrenia-related behavior in *Disc1* mutant mice with increased LCN2 and GFAP^+^ glial cells in the SVZ. We found similar results with pharmacological inhibition (Figure 4a–d) and genetic inactivation of *Lcn2* (Figure 5c–d; **Table S8**). LCN2 is a glycoprotein initially purified from neutrophils [55], that transports fatty acids and iron, induces apoptosis, regulates innate immunity [30], and is a pro-neoplastic factor [30]. LCN2 is secreted by astrocytes [56] and over-expression in zebrafish increased the number and activity of GFAP^+^ glial cells [34]. Furthermore, mild cognitive impairment is associated with higher plasma LCN2 levels [57]. One possible connection between *Disc1* and *Lcn2* is the Rho-ROCK pathway, through which LCN2 modifies reactive astrocytes [34]. The *Disc1*-L100P mutation decreased expression of three Rho-related genes (*Cdc42ep2; Arhgap24* and *Rock2*; **Table S3**).

There are several limitations of the work described here. A recent study of the *Disc1*-L100P mice backcrossed onto the C57BL/6JJcl strain did not show the same phenotype as the original paper characterizing this mutant [11], [58]. C57BL/6JJcl was the original strain subject to ENU mutagenesis, but C57BL/6J was the strain backcrossed before phenotyping in the original report. Thus it is possible that genetic background could affect the phenotype, and that the results of the cross to the *Lcn2* knock-out mice are not specific to inactivation of the *Lcn2* gene. Previous studies have found that the antipsychotics haloperidol [13] and clozapine [11] corrected behavioural abnormalities in adult (week 12–16) *Disc1*-L100P mice, but did not assess effects on behaviour when treatment was delivered earlier, nor whether the drug effects persisted after treatment stopped, as was done in the current study. Finally, the L100P (T334C) mutation is not a polymorphism in humans associated with schizophrenia, so direct comparison across species is not possible. However, the *Disc1*-L100P mutation does provide general insights about the possible effects of *DISC1* variants in humans.

In conclusion, the current study demonstrates that *Disc1*-L100P mutant mice have a developmental onset of behavioral and cellular abnormalities that parallels some clinical features of schizophrenia. We have shown that glial proliferation is increased by *Disc1*-L100P mutation and that early treatment with valproic acid or by genetic inactivation of *Lcn2* can rectify this abnormal proliferation in conjunction with normalizing behavior. Future experiments will investigate molecular mechanisms underlying the functional association of DISC1 and LCN2 in the brain and the potential for valproic acid as a treatment in prodromal patients. LCN2 might also be useful as new biomarker for the prodromal stage of schizophrenia and as a potential new therapeutic target.

## Supporting Information

Table S1 **Acoustic Startle Response in Vehicle−/Valproic acid-treated** ***Disc1*** **-L100P and WT mice.**
(DOCX)

Table S2 **List of up-regulated genes affected by** ***Disc1*** **-L100P mutation in the hippocampus.**
(DOCX)

Table S3 **List of down-regulated genes affected by** ***Disc1*** **-L100P mutation in the hippocampus.**
(DOCX)

Table S4 **List of genes affected by** ***Disc1*** **-L100P mutation in the brainstem.**
(DOCX)

Table S5 **List of genes affected by valproic acid in a** ***Disc1*** **-independent manner.**
(DOCX)

Table S6 **List of genes affected by** ***Disc1*** **-L100P × valproate interactions.**
(DOCX)

Table S7 **Immunohistochemistry for neuron number, cell death and cell proliferation.**
(DOCX)

Table S8 **Effects of genetic inactivation of** ***Lcn2*** **on intensity of GFAP^+^ and LCN2^+^ cells per um^2^ in the brain of 12 week old** ***Disc1*** **-L100P mutant mice.**
(DOCX)
