# Supplementary material for: Prophylactic Valproic Acid Treatment Prevents Schizophrenia-Related Behaviour in Disc1-L100P Mutant Mice
Source: PLoS One. 2012 Dec 18;7(12):e51562. doi: 10.1371/journal.pone.0051562 (PMC3525594; doi:10.1371/journal.pone.0051562)
Supplement: Table S1 — Acoustic Startle Response in Vehicle−/Valproic acid-treated Disc1 -L100P and WT mice. (DOCX) [file pone.0051562.s001.docx]

**Table S1.** Acoustic Startle Response in Vehicle-/Valproic acid-treated *Disc1*-L100P and WT mice

| **Genotype/Drug** | **Vehicle** | **Valproate** |
| --- | --- | --- |
| **20 hours after the last injection of Valproate** | | |
| **Acoustic Startle Response** | | |
| WT (n=16/18) | 920.1 ± 89.1 | 943.6 ± 142.8 |
| DISC1-L100P (n=29/36) | 512.1 ± 150.2 * | 676.3 3 ± 107.1 # |
| **3 weeks after the last injection of Valproate** | | |
| **Acoustic Startle Response** | | |
| WT (n=11/12) | 833.6 ± 57.7 | 827.6 ± 66.8 |
| *Disc1*-L100P (n=10/11) | 447.8 ± 58.6 * | 716.5 ± 82.9 # |

*p<0.001 in comparison with vehicle-treated WT mice; #p<0.05 in comparison with vehicle-treated *Disc1*-L100P mice; ANOVA detected effect of genotype [F_1,95_ = 32.4, p<0.001] but no effect of valproate [F_1,95_ = 4.7, p>0.05] on startle response after 20 hours after the last injection of valproate; ANOVA detected effect of the drug treatment [F_1,40_ = 3.87, p≤0.05] and a nearly significant effect of the gene x drug interaction [F_1,40_ = 4.4, p=0.07] on ASR assessed 3 weeks after the last injection of valproate.
