# Supplementary material for: Prophylactic Valproic Acid Treatment Prevents Schizophrenia-Related Behaviour in Disc1-L100P Mutant Mice
Source: PLoS One. 2012 Dec 18;7(12):e51562. doi: 10.1371/journal.pone.0051562 (PMC3525594; doi:10.1371/journal.pone.0051562)
Supplement: Table S2 — List of up-regulated genes affected by Disc1 -L100P mutation in the hippocampus. (DOCX) [file pone.0051562.s002.docx]

**Table S2.** List of up-regulated genes affected by *Disc1*-L100P mutation in the hippocampus

| **Gene symbol** | **Gene name**  **NCBI ID** | **Function** | **p-Value** |
| --- | --- | --- | --- |
| Pglyrp1 | Peptidoglycan recognition protein 1  **21946** | Immune system; Apoptosis | 9.94E-03 |
| Gga1 | Golgi associated, gamma adaptin ear containing, ARF binding protein 1  **106039** | Vesicle-mediated transport, Intracellular protein transport | 5.26E-02 |
| Upf1 | UPF1 regulator of nonsense transcripts homolog (yeast)  **19704** | mRNA catabolic process | 7.46E-02 |
| Pacsin1 | Protein kinase C and casein kinase substrate in neurons 1  **23969** | Cytoskeleton, Endocytosis | 5.03E-02 |
| Purb | Purine rich element binding protein B  **19291** | Transcriptional factor, Apoptosis, Proliferation | 3.03E-02 |
| Stard13 | StAR-related lipid transfer (START) domain containing 13  **243362** | Cytoskeleton, Cell cycle, Proliferation, Signal transduction | 5.25E-02 |
| A930018M24Rik | RIKEN cDNA A930018M24 gene  **328399** | Unknown | 5.26E-02 |
| Ppfia3 | protein tyrosine phosphatase, receptor type, f polypeptide (PTPRF), interacting protein (liprin), alpha 3  **76787** | Unknown | 6.02E-02 |
| Lcn2 | Lipocalin 2 **16819** | Immune system, Proliferation, Apoptosis | 7.24E-02 |
| Rnase4 | Ribonuclease, RNase A family 4  **16819** | Catalyzes the degradation of RNA | 7.72E-02 |
| Celf4 | CUGBP, Elav-like family member 4  **108013** | mRNA binding and splicing | 8.99E-02 |
| Slc35c2 | Solute carrier family 35, member C2  **228875** | Transport | 8.99E-02 |
| Caskin1 | CASK interacting protein 1  **268932** | Signal transduction | 9.07E-02 |
| Lrrc8a | Leucine rich repeat containing 8A  **241296** | Unknown | 9.11E-02 |
| Xrcc1* | X-ray repair complementing defective repair in Chinese hamster cells 1  **22594** | DNA repair | 9.11E-02 |
| Agxt2l1* | *Alanine-glyoxylate aminotransferase 2-like 1*  **71760** | Unknown | 9.75E-02 |
| Adar | Adenosine deaminase, RNA-specific  **56417** | Transcriptional factor, Apoptosis, Proliferation, cell migration | 9.75E-02 |
| Eif4ebp2 | Eukaryotic translation initiation factor 4E binding protein 2  **13688** | Translation; Synaptic plasticity and memory | 9.9E-02 |

Expression of genes corrected by valproate are highlighted; *genes associated with schizophrenia
