# Supplementary material for: Prophylactic Valproic Acid Treatment Prevents Schizophrenia-Related Behaviour in Disc1-L100P Mutant Mice
Source: PLoS One. 2012 Dec 18;7(12):e51562. doi: 10.1371/journal.pone.0051562 (PMC3525594; doi:10.1371/journal.pone.0051562)
Supplement: Table S3 — List of down-regulated genes affected by Disc1 -L100P mutation in the hippocampus. (DOCX) [file pone.0051562.s003.docx]

**Table S3.** List of down-regulated genes affected by *Disc1*-L100P mutation in the hippocampus

| **Gene symbol** | **Gene name**  **NCBI ID** | **Function** | **p-Value** |
| --- | --- | --- | --- |
| Arc | *Activity regulated cytoskeletal-associated protein*  **11838** | Cytoskeleton, Synapse development, Synaptic plasticity and memory | 5.26E-02 |
| Egr2 | Early growth response 2 **13654** | Transcriptional Factor, Myelination, Motor axon guidance, Brain segmentation; Apoptosis, Immune system, Cognition | 2.46E-02 |
| Xpo7 | Exportin 7 **65246** | Transport; Proliferation, Cell division via export of 14-3-3σ | 1.64E-02 |
| Dusp1 | Dual specificity phosphatase 1 **19252** | Apoptosis, Proliferation | 2.46E-02 |
| Fam63a | *Family with sequence similarity 63, member A*  **75007** | Cell differentiation | 4.27E-02 |
| Cdc42ep2 | *CDC42 effector protein (Rho GTPase binding) 2*  **104252** | Cytoskeleton, cell shape | 7.46E-02 |
| Slc40a1 | Solute carrier family 40 (iron-regulated transporter), member 1 **53945** | Iron ion transmembrane transporter | 7.24E-02 |
| Arhgap24 | Rho GTPase activating protein 24 **231532** | Proliferation, Cell morphology, motility, aggregation, differentiation; Angiogenesis | 7.46E-02 |
| BC066028 | *cDNA sequence BC066028*  **407812** | Metal ion binding | 4.96E-02 |
| Egr4 | Early growth response 4 **13656** | Transcriptional Factor, Synaptic plasticity | 5.26E-02 |
| Ddah1 | *Dimethylarginine dimethylaminohydrolase 1*  **69219** | Arginine metabolism, Nitric oxide biosynthesis; Angiogenesis | 5.26E-02 |
| Rnf214 | Ring finger protein 214 **235315** | Metal ion binding; protein binding | 5.26E-02 |
| Slc35c1 | Solute carrier family 35, member C1 **228368** | Lipid glycosylation | 5.26E-02 |
| Ssb4 | *splA/ryanodine receptor domain and SOCS box containing 4*  **211949** | Intracellular signaling pathway | 6.00E-02 |
| BC005624 | *cDNA sequence BC005624*  **227707** | Unknown | 6.00E-02 |
| 1700123O20Rik | RIKEN cDNA 1700123O20 gene **58248** | Unknown | 6.00E-02 |
| Ppp2ca* | *Protein phosphatase 2 (formerly 2A), catalytic subunit, alpha isoform*  **19052** | Proliferation, Cell cycle | 6.00E-02 |
| Junb | Jun-B oncogene **16477** | Transcriptional Factor, Proliferation, Cell cycle, Vasculogenesis | 6.74E-02 |
| Lrsam1 | *Leucine rich repeat and sterile alpha motif containing 1*  **227738** | Metal ion binding, Protein transport | 6.74E-02 |
| Dcun1d4 | *DCN1, defective in cullin neddylation 1, domain containing 4*  **100737** | Unknown | 7.24E-02 |
| Fntb | Farnesyltransferase, CAAX box, beta **110606** | Proliferation, Cell cycle, Immune system | 7.24E-02 |
| Rpf1 | *Ribosome production factor 1 homolog (S. cerevisiae)*  **70285** | Ribosome biogenesis, rRNA processing | 7.46E-02 |
| Aplp2 | *Amyloid beta (A4) precursor-like protein_2_*  **11804** | Embryonic development, Synaptic transmission, Apoptosis | 7.46E-02 |
| Igf1 | Insulin-like growth factor 1 **16000** | Proliferation, Apoptosis, Cell development cell differentiation, migration | 7.46E-02 |
| Irf2bp1 | *Interferon regulatory factor 2 binding protein 1*  **272359** | Transcriptional Factor; Proliferation | 7.46E-02 |
| Elmo1 | *Engulfment and cell motility 1, ced-12 homolog (C. elegans)*  **140580** | Cytoskeleton, Cell motility, Apoptosis | 7.46E-02 |
| Kbtbd4 | *Kelch repeat and BTB (POZ) domain containing 4*  **67136** | Unknown | 7.46E-02 |
| Fam185a | *Family with sequence similarity 185, member A*  **330050** | Unknown | 7.46E-02 |
| Smap2 | *Stromal membrane-associated GTPase-activating protein 2*  **69780** | Metal ion binding, GTPase activity | 7.46E-02 |
| Samd4 | Sterile alpha motif domain containing 4 **74480** | Post-translational regulator in neurons at post-synaptic level | 7.46E-02 |
| Mtap7d1 | *Microtubule-associated protein 7 domain containing 1*  **245877** | Cytoskeleton | 7.72E-02 |
| Rpap3 | RNA polymerase II associated protein 3 **71919** | Unknown | 8.88E-02 |
| Dnajc6 | *DnaJ (Hsp40) homolog, subfamily C, member 6*  **72685** | Endocytosis | 8.99E-02 |
| Csrnp2 | Cysteine-serine-rich nuclear protein 2 **207785** | Transcriptional Factor, Apoptosis | 8.99E-02 |
| Nup210 | Nucleoporin 210 **54563** | mRNA transport, protein transport | 9.11E-02 |
| Rasl12 | RAS-like, family 12 **70784** | Signal transduction, GTPase activity | 9.11E-02 |
| Rnft2 | Ring finger protein, transmembrane 2 **269695** | Unknown | 9.26E-02 |
| Tomm20 | *Translocase of outer mitochondrial membrane 20 homolog (yeast)*  **67952** | Unfolded protein binding, Protein transport | 9.26E-02 |
| 1200014J11Rik | RIKEN cDNA 1200014J11 gene **66874** | Unknown | 9.62E-02 |
| Fhl1 | Four and a half LIM domains 1 **14199** | Multicellular organismal development, Cell differentiation, Apoptosis | 9.62E-02 |
| Ceecam1 | *Cerebral endothelial cell adhesion molecule*  **99151** | Cell adhesion | 9.75E-02 |
| Ang1 | *Angiopoietin 1*  **11600** | Angiogenesis, Apoptosis, Cell adhesion, differentiation, Multicellular organismal development | 9.75E-02 |
| Phc1 | *Polyhomeotic-like 1 (Drosophila)*  **13619** | Chromatin binding activity, Multicellular organismal development | 9.75E-02 |

Expression of genes corrected by valproate are highlighted; *genes associated with schizophrenia
