# Supplementary material for: Prophylactic Valproic Acid Treatment Prevents Schizophrenia-Related Behaviour in Disc1-L100P Mutant Mice
Source: PLoS One. 2012 Dec 18;7(12):e51562. doi: 10.1371/journal.pone.0051562 (PMC3525594; doi:10.1371/journal.pone.0051562)
Supplement: Table S4 — List of genes affected by Disc1 -L100P mutation in the brainstem. (DOCX) [file pone.0051562.s004.docx]

**Table S4.** List of genes affected by *Disc1*-L100P mutation in the brainstem

| **Gene Symbol** | **Gene name**  **NCBI ID** | **Functions** | **P-values** |
| --- | --- | --- | --- |
| Mrpl39 | mitochondrial ribosomal protein L39  27393 | Mitochondrial genome maintenance, Translation | 7.08E-02 |
| Jph2 | junctophilin 2  59091 | Elevation of cytosolic Ca2+, multicellular organismal development | 7.08E-02 |
| Wnt6 | wingless-related MMTV integration site 6  22420 | Multicellular organismal development, Cell-cell signaling, Signal transduction, organ morphogenesis | 7.08E-02 |
| Klrg1 | killer cell lectin-like receptor subfamily G, member 1  50928 | Immune system, cell surface receptor linked signaling pathway | 7.08E-02 |
| Kcnq2 | potassium voltage-gated channel, subfamily Q, member 2  16536 | Potassium ion transport, Seizures, hyperactivity | 7.08E-02 |
| Slc6a12 | solute carrier family 6 (neurotransmitter transporter, betaine/GABA), member 12  14411 | Betaine / Carnitine / Choline transporter, Cellular water homeostasis, Neurotransmitter transport | 7.26E-02 |
| Chrna9 | cholinergic receptor, nicotinic, alpha polypeptide 9  231252 | Cation transport, Sensory detection of sound and inner ear morphogenesis | 7.26E-02 |
| Sphk1 | sphingosine kinase 1  20698 | Cell differentiation, motility, Apoptosis | 8.91E-02 |
| Ranbp3l | RAN binding protein 3-like  223332 | Intracellular transport | 8.91E-02 |
| Mrgprf | MAS-related GPR, member F  211577 | Signal transduction [G-protein coupled receptor activity) | 8.91E-02 |
| Upk1a | uroplakin 1A  109637 | Endocytosis, Signal transduction | 8.91E-02 |
| Lcn2* | Lipocalin 2 16819 | Immune system, Proliferation, Apoptosis | 7.08E-02 |
| Ly6c* | lymphocyte antigen 6 complex, locusC1  17067 | Immune system | 8.91E-02 |

Non-marked genes are down-regulated; *up-regulated genes; Expression of genes corrected by valproate are highlighted
