# Supplementary material for: Prophylactic Valproic Acid Treatment Prevents Schizophrenia-Related Behaviour in Disc1-L100P Mutant Mice
Source: PLoS One. 2012 Dec 18;7(12):e51562. doi: 10.1371/journal.pone.0051562 (PMC3525594; doi:10.1371/journal.pone.0051562)
Supplement: Table S5 — List of genes affected by valproic acid in a Disc1 -independent manner. (DOCX) [file pone.0051562.s005.docx]

**Table S5.** List of genes affected by valproic acid in a Disc1-independent manner

| **Gene Symbol** | **Gene name**  **NCBI ID** | **Functions** | **P-values** |
| --- | --- | --- | --- |
| Hippocampus | | | |
| Hist1h1c | Histone cluster 1, H1c **50708** | Nucleosome Assembly, Epigenetic | 4.36E-04 |
| Hist1h2be | Histone cluster 1, H2be **319179** | Nucleosome Assembly, Epigenetic | 1.13E-03 |
| Clcn2 | Chloride channel 2 **12724** | Neuronal conductance/excitability | 8.3E-02 |
| Striatum | | | |
| Egr2 | Early growth response 2 **13654** | Transcriptional Factor, Myelination, Motor axon guidance, Brain segmentation; Apoptosis, Immune system, Cognition | 5.54E-02 |
| Fosb | FBJ osteosarcoma oncogene B **14282** | Transcriptional factor, Cell proliferation, differentiation | 5.54E-02 |
