# Supplementary material for: Prophylactic Valproic Acid Treatment Prevents Schizophrenia-Related Behaviour in Disc1-L100P Mutant Mice
Source: PLoS One. 2012 Dec 18;7(12):e51562. doi: 10.1371/journal.pone.0051562 (PMC3525594; doi:10.1371/journal.pone.0051562)
Supplement: Table S6 — List of genes affected by Disc1 -L100P × valproate interactions. (DOCX) [file pone.0051562.s006.docx]

**Table S6.** List of genes affected by *Disc1*-L100P x valproate interactions

| **Gene Symbol** | | **Gene name & NCBI ID** | **Functions** | **P-values**  **( G x D)** |
| --- | --- | --- | --- | --- |
| **Up-regulated genes** | | | | |
| Irx3 | *Iroquois related homeobox 3 (Drosophila)*  **16373** | | Transcriptional factor, Neurodevelopment, Neuronal differentiation | 2.99E-02 |
| Zfp36l1 | *zinc finger protein 36, C3H type-like 1*  **12192** | | Early-response transcription factor, regulates the response to growth factors | 4.17E-02 |
| Mcam | *melanoma cell adhesion molecule*  **84004** | | Endothelial junction associated with the actin cytoskeleton | 5.81E-02 |
| Usp29 | *Ubiquitin specific peptidase 29*  **57775** | | Ubiquitin-dependent protein catabolic process, Epigenetic | 7.09E-02 |
| Nxf* | *NPAS4 neuronal PAS domain protein 4*  **266743** | | Transcriptional factor | 8.05E-02 |
| Cyp27a1* | CYP27A1 cytochrome P450, family 27, subfamily A, polypeptide 1  **1593** | | Catalyze many reactions involved in drug metabolism, synthesis of cholesterol, steroids and other lipids | 8.14E-02 |
| P2ry13 | *Purinergic receptor P2Y, G-protein coupled 13*  **74191** | | Apoptosis | 8.54E-02 |
| Gab1 | *Growth factor receptor bound protein 2-associated protein 1*  **14388** | | Proliferation, Cytoskeleton, Apoptosis | 9.01E-02 |
| **Down-regulated genes** | | | | |
| Ntrk2* | *Neurotrophic tyrosine kinase, receptor, type 2*  **18212** | | Neurodevelopment, dendritic branching, synaptic connections, regulator of glial cells, Apoptosis, Epigenetic | 2.99E-02 |
| Gna11* | *Guanine nucleotide binding protein, alpha 11*  **14672** | | Genetic interaction with dopamine D_2_ receptors, D_1A_ receptors | 3.75E-02 |
| Rock2 | *Rho-associated coiled-coil containing protein kinase 2*  **19878** | | Apoptosis, Proliferation, Axonal growth, Cytoskeleton | 8.71E-02 |
| Bai2 | *Brain-specific angiogenesis inhibitor 2*  **230775** | | Transcriptional Factor, Immune system, Angiogenesis | 9.13E-02 |
| Bicd2 | *Bicaudal D homolog 2 (Drosophila)*  **76895** | | Cytoskeleton | 9.25E-02 |
| Dsip1* | *Delta sleep-inducing peptide Tsc22d3 TSC22 domain family, member 3*  **14605** | | Endocrine regulator, affect physiological functions (temperature, blood pressure, sleep pattern, stress-limiting factor), anti-carciogenic, anti-convulsant | 9.57E-02 |

* - genes, associated with schizophrenia
