# Supplementary material for: Prophylactic Valproic Acid Treatment Prevents Schizophrenia-Related Behaviour in Disc1-L100P Mutant Mice
Source: PLoS One. 2012 Dec 18;7(12):e51562. doi: 10.1371/journal.pone.0051562 (PMC3525594; doi:10.1371/journal.pone.0051562)
Supplement: Table S7 — Immunohistochemistry for neuron number, cell death and cell proliferation. (DOCX) [file pone.0051562.s007.docx]

**Table S7.** Immunohistochemistry for neuron number, cell death and cell proliferation.

**A.** Number of GFAP^+^ and NeuN^+^ cells per mm^2^ in the brains of 12 week-old *Disc1-*L100P and WT mice

| **Brain area** | **WT (n = 16)** | ***Disc1*-L100P (n = 16)** |
| --- | --- | --- |
| **GFAP^+^ cells** | | |
| Olfactory bulbs | 1183.2 ± 211.5 | 1393.5 ± 161.3 * |
| Rostral migratory stream | 1168.2 ± 209.6 | 1358.7 ± 186.4 * |
| Subgranular zone | 625.6 ± 199.4 | 732.9 ± 208.7 |
| **NeuN^+^ cells** | | |
| Olfactory bulbs | 807.8 ± 58.4 | 870.8 ± 72.7 |
| Rostral migratory stream | 200.5 ± 35.5 | 250.5 ± 33.9 |
| Subgranular zone | 1698.4 ± 204.9 | - 1. ± 264.8 |

* - p < 0.05 – in comparison with WT mice. ANOVA detected a significant effect of genotype on amount of GFAP^+^ cells in **OB** [F_1,30_ = 7.71; p<0.01], **RMS** [F_1,30_ = 8.18, p<0.01], but not in **SGZ** [F_1,30_ = 0.39, p>0.05].

**B.** Number of TUNEL-positive cells per mm^2^ in the brains of 12 week-old *Disc1*-L100P and WT mice

| **Brain area** | **WT (n = 16)** | **Disc1-L100P (n = 16)** |
| --- | --- | --- |
| Olfactory bulbs | 2.4 ± 1.0 | 1.4 ± 0.4 |
| Rostral migratory stream | 2.6 ± 0.5 | 1.3 ± 0.3 |
| Subventricular zone | 2.3 ± 0.5 | 1.9 ± 0.3 |
| Subgranular zone | 1.4 ± 0.2 | 1.2 ± 0.3 |

**C.** Number of Ki67-positive cells per mm^2^ in the brains of 8 week-old *Disc1*-L100P and WT mice

| **Brain area** | **WT (n = 18)** | **DISC1-L100P (n = 17)** |
| --- | --- | --- |
| Olfactory bulbs | 22.9 ± 8.5 | 18.7 ± 4.9 |
| Rostral migratory stream | 24.6 ± 7.5 | 18.4 ± 6.9 |
| Subventricular zone | 12.3 ± 1.7 | 20.8 ± 8.2 |
| Subgranular zone | 23.4 ± 8.7 | 16.2 ± 3.0 |
