# Supplementary material for: Prophylactic Valproic Acid Treatment Prevents Schizophrenia-Related Behaviour in Disc1-L100P Mutant Mice
Source: PLoS One. 2012 Dec 18;7(12):e51562. doi: 10.1371/journal.pone.0051562 (PMC3525594; doi:10.1371/journal.pone.0051562)
Supplement: Table S8 — Effects of genetic inactivation of Lcn2 on intensity of GFAP+ and LCN2+ cells per um2 in the brain of 12 week old Disc1 -L100P mutant mice. (DOCX) [file pone.0051562.s008.docx]

**Table S8.** Effects of genetic inactivation of *Lcn2* on intensity of GFAP^+^ and LCN2^+^ cells per um^2^ in the brain of 12 week old *Disc1*-L100P mutant mice

| **Genotype/ Brain Area** | | **Olfactory Bulbs** | | **Rostral Migratory Stream** | | **Subgranular zone** | |
| --- | --- | --- | --- | --- | --- | --- | --- |
| **DISC1** | **LCN2** | **GFAP** | **LCN2** | **GFAP** | **LCN2** | **GFAP** | **LCN2** |
| WT | WT | 630.6±73.3 | 358.4±44.0 | 585.5±96.1 | 645.3±48.2 | 574.0±91.4 | 394.8±86.4 |
| L100P | WT | 858.0±90.2* | 674.5±73.9* | 890.1±98.1* | 1048.7±33.3** | 398.8±99.1 | 434.0±61.9 |
| WT | LCN2-KO | 408.4±90.4 | 0 | 392.5±88.5 | 0 | 417.3±97.7 | 0 |
| L100P | LCN2-KO | 613.6±93.1 | 0 | 593.5±83.7 | 0 | 612.8±89.6 | 0 |

* - p < 0.05; ** - p < 0.01 – in comparison with WT/WT mice; unpaired *t-test.* (N = 4 slices per mouse; 3-5 mice per genotype).
